# Spiral cleavage and early embryology of a loxosomatid entoproct and the usefulness of spiralian apical cross patterns for phylogenetic inferences

**DOI:** 10.1186/1471-213X-12-11

**Published:** 2012-03-29

**Authors:** Julia Merkel, Tim Wollesen, Bernhard Lieb, Andreas Wanninger

**Affiliations:** 1 Johannes Gutenberg University, Institute of Zoology, 55099 Mainz, Germany; 2 University of Vienna, Dept. of Integrative Zoology, Althanstrasse 14, 1090 Vienna, Austria

**Keywords:** Lophotrochozoa, Embryology, Development, Ontogeny, Evolution, Phylogeny, Spiral cleavage, Molluscan cross, Annelid cross

## Abstract

**Background:**

Among the four major bilaterian clades, Deuterostomia, Acoelomorpha, Ecdysozoa, and Lophotrochozoa, the latter shows an astonishing diversity of bodyplans. While the largest lophotrochozoan assemblage, the Spiralia, which at least comprises Annelida, Mollusca, Entoprocta, Platyhelminthes, and Nemertea, show a spiral cleavage pattern, Ectoprocta, Brachiopoda and Phoronida (the Lophophorata) cleave radially. Despite a vast amount of recent molecular phylogenetic analyses, the interrelationships of lophotrochozoan phyla remain largely unresolved. Thereby, Entoprocta play a key role, because they have frequently been assigned to the Ectoprocta, despite their differently cleaving embryos. However, developmental data on entoprocts employing modern methods are virtually non-existent and the data available rely exclusively on sketch drawings, thus calling for thorough re-investigation.

**Results:**

By applying fluorescence staining in combination with confocal microscopy and 3D-imaging techniques, we analyzed early embryonic development of a basal loxosomatid entoproct. We found that cleavage is asynchronous, equal, and spiral. An apical rosette, typical for most spiralian embryos, is formed. We also identified two cross-like cellular arrangements that bear similarities to both, a "molluscan-like" as well as an "annelid-like" cross, respectively.

**Conclusions:**

A broad comparison of cleavage types and apical cross patterns across Lophotrochozoa shows high plasticity of these character sets and we therefore argue that these developmental traits should be treated and interpreted carefully when used for phylogenetic inferences.

## Background

Currently, bilaterian animals are subdivided into four major groups: the supposedly basal Acoelomorpha, the Ecdysozoa (combining all molting animals such as arthropods and nematodes), the Lophotrochozoa with a trochophore-like ciliated larva (e.g., Annelida, Entoprocta, Mollusca, Platyhelminthes), and Deuterostomia (including chordates, hemichordates and echinoderms) [1-4]. Despite ongoing efforts, the interrelationships of the phyla that nest within the Lophotrochozoa remain unresolved [5,6]. Entoprocta is a phylum that has been proposed to belong to a clade of spirally cleaving animals, the so-called Spiralia, which together with its suggested sister group, the Lophophorata (Ectoprocta, Brachiopoda, and Phoronida), forms the Lophotrochozoa [1]. Typically, entoprocts are microscopic, mostly marine, sessile metazoan animals. Its approximately 150 hitherto described species are divided into four subgroups, the solitary (and supposedly basal) Loxosomatidae and the colonial Barentsiidae, Pedicellinidae, and Loxocalypodidae [7]. Their adult gross morphology is characterized by a ciliated tentacle crown, which surrounds both the mouth and the anus. The calyx houses the reproductive organs, mostly one pair of protonephridia, and the cerebral ganglion. Entoprocts reproduce asexually by budding, as well as sexually, whereby two major larval types can be recognized, namely the creeping, supposedly basal, lecithotrophic and the more common swimming, planktotrophic larval type [8]. Metamorphosis is very complex and often involves settlement and adhesion with the frontal body region to the substrate as well as rotation of the gut [9].

Morphological and molecular analyses have proposed several phylogenetic hypotheses concerning entoproct interphyletic relationships. Traditionally, Entoprocta and Ectoprocta have been comprised to form the monophyletic Bryozoa (Bryozoa-concept), based on a metamorphosing larval stage with a completely retracted and cavity-enclosed prototroch as well as additional common features during metamorphosis [8,9]. This hypothesis has been revived by a recent molecular study [10], although subsequent analyses of partly the same authors are far less clear [11]. The cryptic Cycliophora, one of the most recently erected phyla [12], have also argued to be associated with Entoprocta and Ectoprocta, notably as a monophyletic assemblage termed "Polyzoa" [13,14], while other authors suggest a sister group relationship of Cycliophora and Entoprocta alone [15,16]. On the contrary, the recently proposed Tetraneuralia-concept has strengthened the so-called Lacunifera- or Sinusoida- hypothesis, suggesting a monophyletic assemblage of Entoprocta and Mollusca based on numerous larval and adult autapomorphies [4,17-19]. Resembling a mosaic of larval and adult molluscan characters, the entoproct creeping-type larva shares a number of morphological traits with the polyplacophoran trochophore, including a highly complex apical organ with eight centrally located flask-shaped and several peripheral cells, as well as a typical molluscan-like tetraneurous condition of longitudinal nerve cords [4,19-21]. Additional shared characters are, among a total set of nine, the distinct creeping foot, a large pedal gland, frontal cirri, and a ventrally intercrossing dorsoventral musculature [4,19,20]. Despite the spiral cleavage pattern, which has traditionally been used to unite polyclad flatworms, nemerteans, annelids, and molluscs as "Spiralia" [22], other developmental characters, such as the cellular arrangement into an "apical cross pattern" during early embryogenesis, have been used to infer protostome interrelationships. For a long time, only two cross patterns had been clearly defined, namely the molluscan and the annelid cross, respectively. Since a seemingly "molluscan-type" cross pattern had also been reported for sipunculans, a close relationship to molluscs was suggested [23]. Recently, additional cross patterns, such as a nemertean cross, have been described [24]. For entoprocts, a spiral cleavage pattern has been mentioned in the literature and is often referred to in textbooks, but its documentation is restricted to only a few sketch drawings [25-27]. Apical cross patterns, which would be expected for a spirally cleaving taxon, have not been reported by these studies.

In order to fill the significant gaps in knowledge concerning entoproct early embryology, we herein describe the development of a representative of the supposedly basal entoproct genus *Loxosomella *by applying immunochemistry and confocal microscopy. Using our detailed description of the early cleavage pattern, we also clarify whether or not a distinct "cross pattern" is present in this species. These data are discussed with those available for other lophotrochozoans in order to assess their suitability for phylogenetic inferences.

## Methods

### Animals and fixation

Populations of an undescribed, brooding loxosomatid entoproct belonging to the genus *Loxosomella *(Claus Nielsen, pers. comm.) were collected in July 2007 from tubes of the maldanid polychaete, *Axiothella rubrocincta*, which inhabits the intertidal mud flats of False Bay, San Juan Island, USA. Up to 20 embryos of all developmental stages can be found in one mother individual. From approximately 100 mother animals, we analyzed four individuals each for the 1-, 2-, 4- to 5-, and 8-cell stages, seven embryos that had between eight and 16 cells, and one 21-cell embryo. Approximately 50 embryos were found at the apical rosette stage and three gastrulae were investigated. Numerous swimming-type larvae - obviously shortly before release - were retrieved, indicating the healthy condition of the adults and their developmental stages. The results obtained were highly consistent among the individuals of each developmental stage. Accordingly, the spiral-type cleavage pattern was found in all embryos investigated, although the shape (spherical versus more oval) varied between individuals.

In order to remove the embryos from the mother animal, the tentacle crown was cut open using insect needles. Prior to fixation, adult individuals carrying embryos were relaxed in 7% MgCl_2 _and removed from the polychaete tubes. Fixation was carried out using a solution of 4% paraformaldehyde (PFA) in 0.1 M phosphate buffered saline (PBS) for 1 h at room temperature. After fixation, the specimens were washed in 0.1 M PBS (3 × 15 min) and stored in 0.1 M PBS containing 0.1% NaN_3 _at 4°C.

A reference specimen, determined by Dr. Claus Nielsen (Copenhagen) is deposited at The Natural History Museum of Denmark, Copenhagen (registration tag ZMUC-ENT-27). In addition, the COI gene of our study specimen was partially sequenced and the 710 bp sequence deposited in GenBank (acc. # JQ614997).

### Immunocytochemistry, data generation, and analysis

After fixation and storage, the embryos were permeabilized in 0.1 M PBS with 0.1% Triton X-100 (PBT) for 1 h. F-actin was labeled using a 1:20 dilution of Alexa Fluor 488 phalloidin (Invitrogen, Molecular Probes, Eugene, OR, USA) in PBT. For nucleic acid staining, 10% DAPI (Invitrogen, Taastrup, Denmark) was added and the samples were incubated for 20-24 h at room temperature. Then, the embryos were washed in 0.1 M PBS (3 × 15 min) and mounted in Fluoromount G (Southern Biotech, Birmingham, AL, USA) on glass slides. 5 μl of DAPI (Invitrogen) was added to the embedding medium in order to enhance the signal strength of the nucleic acid staining. The samples were examined using a Leica DM RXE 6 TL fluorescence microscope equipped with a TCS SP2 AOBS laserscanning device (Leica Microsystems, Wetzlar, Germany). The optical sections had a Z-step size of 0.45-0.55 μm. Resulting stacks were merged into projection images with greater focal depth. 3D reconstructions were generated from the confocal datasets using the image processing software Imaris 5.7.2 (Bitplane AG, Zürich, Switzerland).

## Results

### General aspects of entoproct reproduction

**Figure 1:**
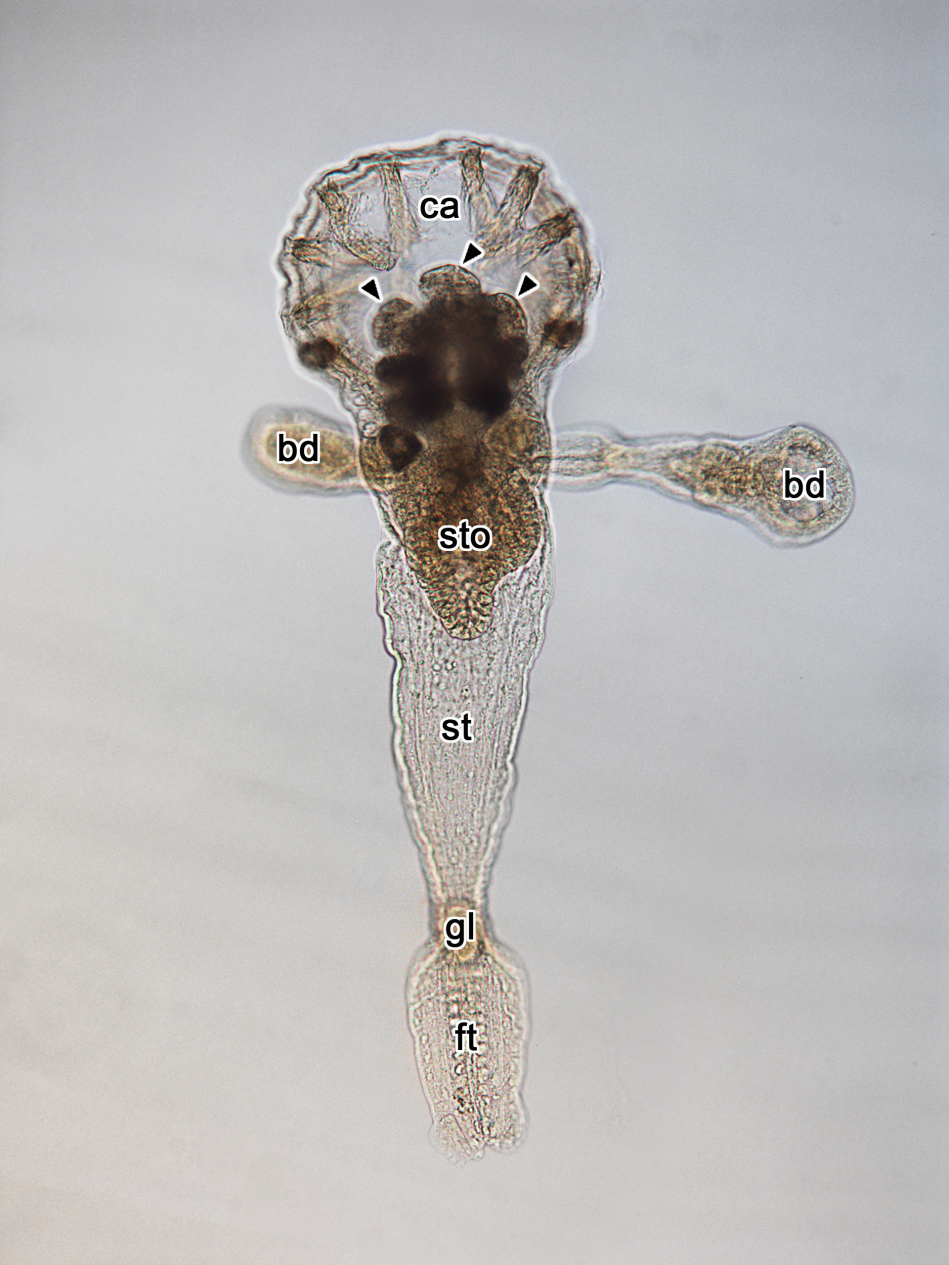
**Brooding *Loxosomella *sp. with embryos (arrowheads) located in the calyx. bd, bud; ca, calyx; ft, foot; gl, foot gland; st, stalk; sto, stomach**. Total length of the animal is approximately 1 mm.

The brood pouch of the loxosomatid species investigated herein is located in the calyx and contains up to 20 embryos (Figure 1). Every embryo is surrounded by a thin membrane. Each membrane tapers in a strand which is connected to the strands of other embryos. Later embryonic stages are located in the anterior region of the brood chamber. The earliest developmental stages are found in the posteriormost part of the brood pouch. Due to the high yolk content, early embryos appear opaque and non-transparent. Released larvae are planktonic and of the swimming-type with a weakly developed foot sole posterior to the prototroch (see [8]).

### Cleavage and gastrulation in *Loxosomella *sp

**Figure 2:**
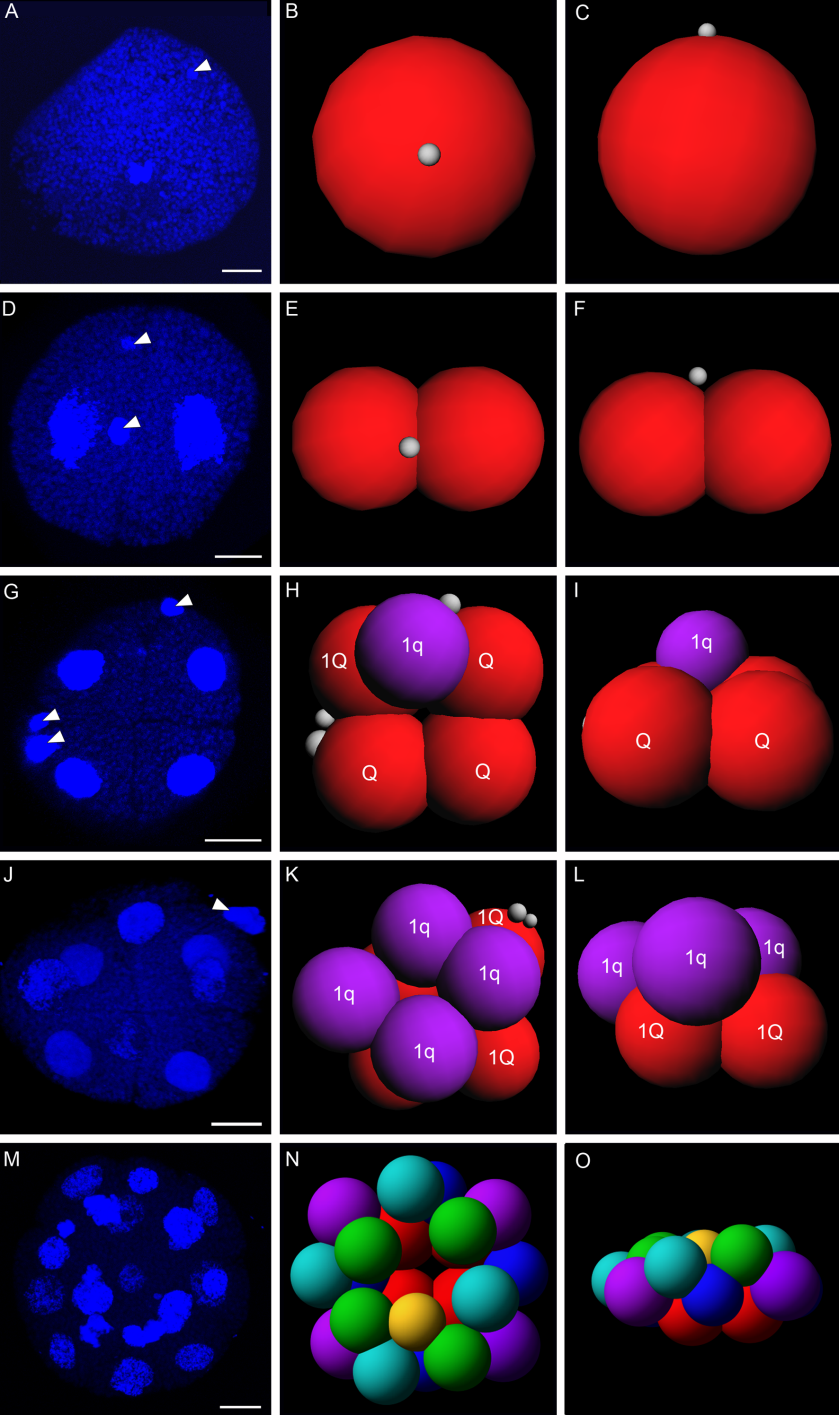
**Confocal micrographs and 3D reconstructions of early cleavage stages of *Loxosomella *sp. Scale bars: 10 μm. left column: Nucleic acid staining (blue)**. **A, C, F, I, L, O**: lateral view; **B, D, E, G, H, J, K, N**: animal view; M: vegetal view. Polar bodies are indicated by arrowheads. Middle and right column: 3D reconstructions, middle column: animal view, right column: lateral view. grey: polar bodies, red: "macromere" quartet cells (nQ cells); purple, blue, light blue, green, yellow: "micromere" quartet cells (nq^n ^cells). **A-C**: Fertilized oocyte prior to first cleavage. One polar body is present on the animal side of the embryo. **D-F**: Two-cell stage. A second polar body appears shifted by 90° relative to the first polar body. The macromeres are equal in size. Second polar body obscured by the red cells in **E **and **F**. **G-I**: Five-cell stage. Three polar bodies are present. Cleavage is asynchronous and the fifth cell (purple) lies between two Q cells (red) in a cleavage furrow. **J-L**: Eight-cell stage. Two polar bodies are present and located next to the vegetal, slightly unequal 1Q cells (red). Each cell of the first "micromere" quartet 1q (purple) is located in a cleavage furrow of two 1Q cells. **M-O**: 21-cell-stage. Cleavage is asynchronous, cells are different in size.

**Figure 3:**
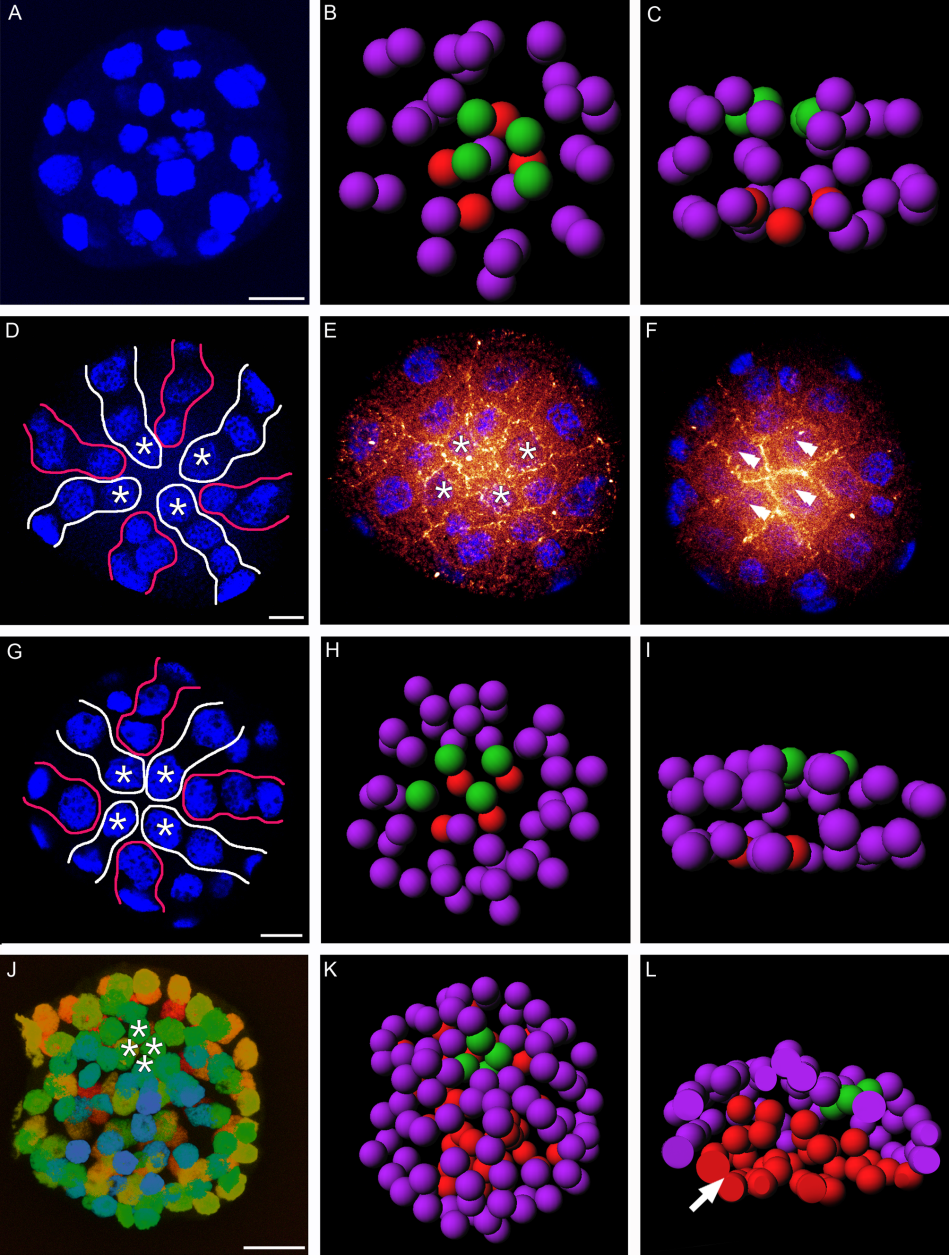
**Confocal micrographs and 3D reconstructions of blastula and gastrula stages of *Loxosomella *sp. "Apical cross patterns" are indicated by white and red lines in D and G**. Apical rosette cell nuclei are marked with asterisks in D, E, G, J and vegetal "macromere" quartet cells by double arrowheads in F. Scale bars: 10 μm. **A, D, G**: Nucleic acid staining (blue). **E, F**: Nucleic acid (blue) and F-Actin staining (red). **J**: Nucleic acid staining shown as depth-coded confocal projection. **A**: vegetal view, **D, E, G, J**: animal view, **F**: vegetal view. **B, C, H, I, K, L**: 3D reconstructions. **B, H, K**: animal view, **C, I, L**: lateral view. red: "macromere" quartet (i.e. vegetal) cell nuclei; green: (derivatives of) apical rosette cell nuclei; purple: other cell nuclei. **A-C**: 36-cell stage. **D-F**: 43-cell stage. **D**: Cells surrounding the apical rosette (asterisks) show both, a molluscan- and an annelid-like cross pattern. **G-I**: 51-cell stage. **J-L**: Gastrula stage (107 cells). **J**: Derivatives of apical rosette cells lie in a lower plane than the surrounding cells. **K, L**: Gastrulation. Vegetal cells (red), blastocoel (arrow).

**Figure 4:**
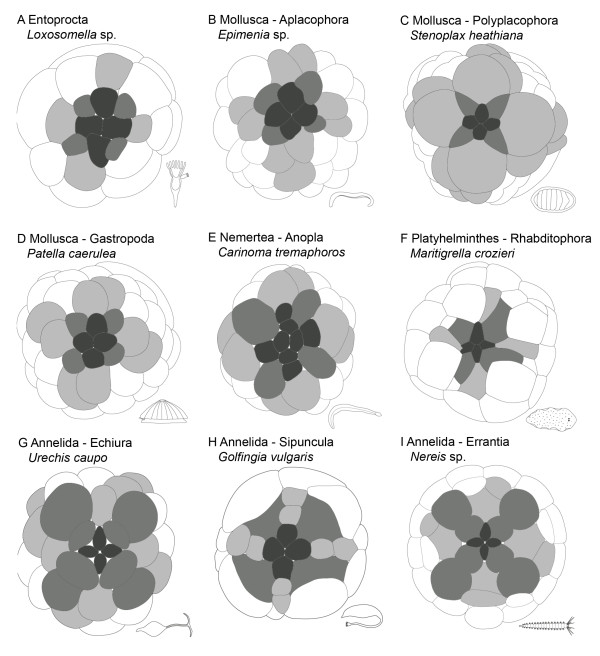
**Spiralian apical cross cell patterns based on several authors as well as the data on *Loxosomella *presented herein**. Dark grey: apical rosette cells, grey: periphere rosette/"annelid cross" cells, light grey: "molluscan cross" cells. **A**: 43-cell stage of *Loxosomella *sp (this study). **B**. Approximately 64-cell stage of the aplacophoran mollusc *Epimenia *sp. (after [48]). **C**: Approximately 62-cell stage of the polyplacophoran mollusc *Stenoplax heathiana *(= *Ischnochiton magdalenensis*; after [32], pl. 2, figure. 17). **D**: 58-cell stage of the gastropod mollusc *Patella caerulea *(after [47], figure. I, 7). **E**: 64-cell stage of the nemertean *Carinoma tremaphoros *(after [24], figure. 6 J). **F**: Approximately 64-cell stage of the polyclad flatworm *Maritigrella crozieri *(after [54], Figure 1 E). G: 64-cell stage of the echiurid *Urechis caupo *(after [55], pl. IV). **H**: 48-cell stage of the sipunculan *Golfingia vulgaris *(= *Phascolosoma vulgare *after [56], pl. XXXII, figure. 9). **I**: Approximately 58-cell stage of the polychaete *Nereis *sp. (after [46], diagram II, B).

Fertilized eggs form a polar body which appears at the animal pole of the embryo (Figure 2A-C). In two-cell stages, both cells are equal in size and show two polar bodies, one on the animal pole and one shifted by approximately 90° relative to the first one (Figure 2D; one polar body is obscured in the 3D reconstructions shown in 2E and F). Second cleavage results in four cells with three polar bodies. Since all cells are approximately equal in size, an assignment of individual cells ("macromeres") to specific quadrants appears difficult. Shortly after second cleavage, a fifth cell (1q) is already present and demonstrates the asynchronicity of early cleavage in our study species (Figure 2G-I). After third cleavage, eight cells have formed and only two polar bodies can be observed. Applying the nomenclature of Conklin (1897) [28], third cleavage results in the "macromere" quartet 1Q and the "micromere" quartet 1q. All cells of the first micromere quartet are equal in size and of approximately the same size as the macromere cells (Figure 2J-L). The blastula of the 21-cell stage is flattened and ellipsoid-shaped. All four macromeres (red cells, Figure 2N, O) are equal in size and nearly twice as large as the four micromeres (blue cells, Figure 2N, O), each situated above the cleavage furrow of two macromeres. A second micromere quartet (light blue cells, Figure 2N, O) is located on top of these micromeres, with similar cell size as the latter (Figure 2M-O). A third quartet of micromeres (purple cells, Figure 2N, O) rests upon the macromeres. Its cells are slightly larger than the micromeres of the other two quartets. A single cell of a fourth micromere quartet (yellow cell, Figure 2N, O) is situated close to the animal pole (Figure 2M-O). At the 36-cell stage, the embryo exhibits four additional, smaller cells, which form an apical rosette on the animal pole. This apical rosette directly faces the macromere quartet on the vegetal side of the embryo (Figure 3B, C). In the 43-cell stage (Figures 3D-F, 4A) and the 51-cell stage (Figure 3G-I), respectively, cells surrounding the apical rosette are arranged in two different cross-shaped patterns (Figures 3D, E, G, 4A). The first cross pattern results from interconnecting rosette cells which lie opposite to each other, resulting in a pattern that closely resembles a molluscan cross. The second pattern is formed by the peripheral rosette cells and appears very similar to a typical "annelid cross" (Figures 3D, E, G, 4A). Overall, cleavage in the *Loxosomella *species investigated herein can be characterized as holoblastic, asynchronous, equal, and spiral. The gastrula stage elongates somewhat along the animal-vegetal axis and shows bilateral symmetry (Figure 3J-L). It consists of approximately 100-110 cells. A blastopore was slightly visible as a small vent on the vegetal side of the 107-cell stage (Figure 3J-L).

## Discussion

Most spiralian lophotrochozoans are characterized by a spiral cleavage pattern, which is mainly defined by (1) the "spiral" arrangement of subsequent embryonic cells (= blastomeres), whereby cells of an upper tier of an embryo come to lie over the cleavage furrow of the lower tier; and (2) formation of a so-called "mesentoblast", which later gives rise to the endomesoderm from the 4 d-cell [7,29]. Despite these overall similarities between spirally cleaving species, the cleavage program is subject to great variability [30], e.g. concerning the size of cells (equal or unequal), regularity (synchronous or asynchronous), direction of cleavage (clockwise vs. counter-clockwise), morphological arrangement of apical cross patterns, or cell fates. In the following, we summarize the various spiralian cleavage phenotypes and discuss them in an evolutionary context in the light of the data presented herein for the entoproct *Loxosomella sp*.

### Cleavage in molluscs

Most molluscs, except for yolk-rich "higher" gastropods and cephalopods, show a more or less "typical" spiral cleavage pattern [31-36]. Cleavage may be equal or unequal. Some species, such as the gastropod *Ilyanassa obsoleta *and the scaphopod *Antalis entalis *(formerly *Dentalium dentale*), form polar lobes which fuse with the D-quadrant of the early embryo [29,33,37]. Although the formation of the first micromere quartet typically appears in a clockwise direction in most taxa, including the supposedly basal solenogaster *Epimenia babai *[35], the polyplacophoran *Stenoplax heathiana *(formerly *Ischnochiton heathiana*; [32]), the scaphopod *Antalis entalis *[33], as well as the gastropods *Limax *[31], *Crepidula *[28,38], *Patella *[39], and *Ilyanassa *[40], a counter-clockwise formation is sometimes found, e.g., in the bivalve *Dreissena polymorpha *[41] or in gastropods with sinistrally coiled shells such as *Planorbis trivolvis *[42], *Physa heterostropha *[43], or *Lymnaea stagnalis *[44]. Accordingly, it appears that in shell-bearing gastropods, the chirality of cleavage is strictly correlated with the direction of shell coiling [43-45]. In contrast to the sinistrally coiled gastropod species mentioned above, fourth cleavage takes place in a counter-clockwise direction [28,29,31,32,35,39].

A typical spiralian feature develops in the following cleavage stages of different taxa, whereby derivatives of the first micromere quartet form the apical (1q^111^) and peripheral rosette (1q^112^). The apical rosette gives rise to the so-called "molluscan cross", whereby its arms are formed by the progenies of the 1q^12^-cells. The tips of the molluscan cross are represented by second micromere quartet cells (2q^1^) and their progenies [28,46]. However, variations in the morphology of the cross pattern occurs among the various molluscan subclades. For example, *Stenoplax heathiana *[32] exhibits relatively large cross cells (1q^121^) as well as pointed peripheral rosette cells which are slightly larger than cells of the apical rosette (Figure 4C). In the basal gastropods *Patella caerulea *[47] and *Patella vulgata *[39], peripheral and apical rosette cells are similar in size and shape and slightly smaller than cells of the arms of the cross (Figure 4D), while a distinct cross pattern was not found in embryos of *Epimenia *[48] (Figure 4B). Cross formation in the scaphopod *Antalis *is characterized by a compressed pentagonal-shaped apical cell 1 d^111^, which contacts the cell 1c^112 ^of the C-arm belonging to the molluscan cross [33]. Although most bivalves do not show a molluscan cross pattern during development [49], a molluscan cross seems to be present in the equal cleaving, basal protobranch *Solemya reidi*, but probably not in the closely related protobranchs *Acila, Nucula, and Yoldia *[50-53]. Accordingly, such a cross pattern may not even be part of the ancestral bivalve bodyplan.

### Cleavage in annelids (including sipunculans)

Embryos of the polychaete *Nereis *[46], the echiuran *Urechis caupo *[55], the sipunculan *Phascolosoma *[56,57], the oligochaete *Bdellodrilus philadelphicus *[58], and the leech *Theromyzon tessulatum *[59] display a holoblastic, spiral cleavage pattern. Cleavage may be equal or unequal. Polar lobe formation is mainly found in polychaetes (e.g. *Hydroides hexagonus*) [60]. The condition of the typical spiralian eight cell stage with small micromeres and large macromeres and a clockwise formation of the first micromere quartet [30] appear to have been modified in various taxa. In the sipunculans *Themiste pyroides *and *Phascolosoma agassizii*, the micromere 1 d is larger than the blastomeres of the A, B, and C quadrant, and the macromere 1D is the largest of all cells [57]. First quartet micromeres are budded off in a clockwise direction [57,61]. In the leech *Theromyzon tessulatum *and the oligochaete *Tubifex rivulorum*, the micromeres of the first quartet are exceptionally small. While the micromeres 1a, c, d are formed in a clockwise direction, 1b is budded off counter- clockwise [59]. For polychaetes, a counter-clockwise formation of first quartet micromeres has so far only been reported for the serpulid *Hydroides elegans *[62]. First quartet micromeres of the echiurid *Urechis caupo *are formed in a clockwise direction [55].

The annelid cross is a seemingly common character of echiurans, clitellates, and polychaetes [63,64], whereby the peripheral rosette cells 1q^112 ^constitute its founding cells [28,46]. However, if 64-cell stages are compared, a typical "annelid cross" is only present in a few taxa such as the echiuran *Urechis caupo *[55] (Figure 4G), the polychaetes *Polygordius *sp. [65], *Amphitrite ornata *[66], and *Nereis *sp. [46] (Figure 4I), as well as the leech *Theromyzon tessulatum *[67]. Annelid cross cells of *Urechis caupo *and *Nereis *sp. are much larger than the apical rosette cells. In both, the more peripherally located cells 1q^1122 ^are slightly larger than their centrally located sister cells 1q^1121 ^(Figure 4G, I). Embryos of *T. tessulatum *show a very indistinct cross pattern. Together with other small micromeres, the annelid cross cells are embedded in the furrows of the comparatively huge macromeres (Figure 494 in [67]). Compared to *Nereis *sp. (Figure 4I), the division of peripheral rosette cells in *Chaetopterus pergamentaceus *is oblique and results in an indistinct cross pattern [66].

The first description of a cross stage in sipunculans was performed on *Phascolosoma vulgare *(= *Golfingia vulgaris*; [56,61]; Figure 4H herein), which is still cited as proof for a molluscan-type cross pattern in a sipunculan [23,68], and thus as an indication for a mollusc-sipunculan sister relationship. However, a number of independent developmental and molecular studies strongly argue in favour of a monophyletic annelid-sipunculan assemblage [10,11,69-75]. Although never explicitly stated for sipunculans, the arrangement of the peripheral rosette cells closely resembles the arrangement of an early stage annelid cross, whereby the sipunculan peripheral rosette cells are considerably larger than their molluscan counterparts (Figure 4). However, convincing recent data on the early embryology of sipunculans are lacking, and thus prevent a final statement as to whether or not an annelid or molluscan cross-like pattern is part of the sipunculan groundplan.

### Cleavage in other lophotrochozoans

Nemertean cleavage is holoblastic, equal, and spiral [24,76,77]. First quartet micromeres of *Cerebratulus lacteus *[78] and *Carinoma tremaphoros *[24] are larger than the macromeres. A so-called "nemertean cross" is found in *Carinoma tremaphoros *([24]; Figure 4E herein) and *Emplectonema gracile *[24]. It is formed by accelerated cell divisions of the apical rosette, which so far has only been reported from nemertean species. Nemertean peripheral rosette cells are relatively large compared to the small apical rosette cells (Figure 4E). Polyclad platyhelminths such as *Maritigrella crozieri *and *Hoploplana inquilina *show a quartet, equal and spiral cleavage pattern [54,79]. In both species, fourth quartet micromeres are very large compared to the relatively small macromeres [54]. Morphologically, the apical and peripheral rosette cells of the 32- to 64-cell stage of the polyclad flatworm *Maritigrella crozieri *could be interpreted as both, a molluscan and an annelid cross pattern, respectively [54]. Although the progenitor cells of the apical and peripheral rosettes have not yet divided at this stage, blastomeres of the 32-cell stage ("third quartet"-stage) of *H. inquilina *form an apical cross-like pattern (see [79,80]). In *Maritigrella crozieri*, peripheral rosette cells are slightly elongated and more than twice as large as the apical rosette cells (Figure 4F).

Developmentally, ectoprocts, phoronids, and brachiopods are unique within the lophotrochozoans because they exhibit a radial cleavage pattern [81-84]. A spiral-type cleavage pattern has been proposed for the phoronid *Phoronopsis viridis *[85] and the brachiopod *Terebratulina septentrionalis *[86] by some classical studies, although these data are highly questioned by recent investigations and may be artifactual due to compression of the embryos [81,85].

### Cleavage in entoprocts

The *Loxosomella *species investigated herein cleaves holoblastic, asynchronous, equal, and spiral, whereby all cells are of approximately the same size until third cleavage. Indicated by the degree of shifting of the micromere cells relative to the macromere cells, the first micromere quartet is formed in a clockwise direction (Figure 2G-L). Accordingly, comparative morphology of the cleavage stages investigated herein clearly shows that our study species exhibits typical spiral cleavage. Following the rule of alternation, in *Loxosomella*, the second quartet micromeres (blue cells, Figure 2N, O) rotate in a counter-clockwise direction until they occupy the furrows between the macromeres (red cells, Figure 2N, O). As a consequence, cells of the first micromere quartet are turned back over the centre of each macromere (cf. [28]). Division of the first micromere quartet results in the 1q^1^- and 1q^2^-quartets (purple cells, Figure 2N, O). The light blue and green colored cells are probably derivatives of the first micromere cells 1q^1^, while the yellow cell is likely to be a derivative of the 1q^11 ^cell (Figure 2N, O). Morphologically, the apical cross pattern in the 43-cell stage of *Loxosomella *sp. exhibits two cross-like patterns, which show similarities to both a "molluscan" and an "annelid cross", respectively (see also [87]), thus supporting closer affinity of Entoprocta with these spiralian taxa than with Ectoprocta. Observations on the colonial entoproct *Pedicellina echinata *(= *Pedicellina cernua*) report both, a slightly unequal [25] and an equal cleavage pattern [26]. Sketch drawings of the first cleavage stages of *Loxosoma leptoclini *were interpreted as showing equally-sized blastomeres [26]. However, since we were not able to trace cell genealogy in our study species, a concluding statement concerning homology between these "entoproct crosses" and the annelid and/or molluscan crosses cannot be given at present. A comparison of the various spiralian-type cleavage patterns, however, does demonstrate the morphological plasticity of the "cross-type" cellular arrangement in the various spiralian representatives, and thus shows that phylogenetic conclusions based on these embryonic morphotypes alone should be treated with utmost care.

## Conclusions

Although it has repeatedly been proposed that the "molluscan cross" constitutes a pattern that is only found in molluscs and closely related sister groups [23,88], similar cross patterns are also present in other spiralian animals including annelids, nemerteans, and flatworms [24,87,89] (cf. Figure 4). The arrangement of the annelid and nemertean cross cells resembles a generation of cells which usually appears during the 7^th ^cleavage cycle, while molluscan cross cells are formed during the 6^th ^division. Since the annelid and nemertean cross is formed earlier in development, the formation of both cross patterns is distinct to these phyla and is not found in 64-cell stages of molluscan embryos. Accordingly, due to their variation even between closely related species, a typical "molluscan" or "annelid" cross pattern cannot reliably be proposed at present, thus rendering these cleavage morphologies phylogenetically uninformative. However, we suggest that an embryonic stage with a cross-like pattern was present in the last common ancestor of Spiralia. The cleavage pattern of the entoproct *Loxosomella *sp. investigated herein shows typical spiralian features, such as the spiral arrangement of blastomeres around the animal-vegetal axis of the embryo and the presence of a cross-like pattern, thus indeed rendering entoprocts "true" spiralians.

The currently widely held view that the radially cleaving Ectoprocta is not the sister group of Entoprocta is well supported by our data and strongly suggests the inclusion of Entoprocta within Spiralia. This is also in agreement with the Tetraneuralia-hypothesis, which suggests a Mollusca-Entoprocta clade [4] and argues against the so-called Polyzoa-concept (Ectoprocta + Entoprocta + Cycliophora; see [14]). The latter scenario would either imply independent evolution of spiral cleavage in Entoprocta and the remaining Spiralia or secondary loss of spiral cleavage in Ectoprocta (data on cleavage in Cycliophora are still lacking). On a deeper evolutionary scale, the classical subdivision of Lophotrochozoa into Spiralia and Lophophorata suggests either a spiral or a radial cleavage pattern for the last common lophotrochozoan ancestor. The duet-spiral-type of cleavage in the supposedly basal bilaterians, the acoels, may favour a scenario where a modification of this cleavage pattern might have resulted in the quartet-spiral cleavage pattern of the ur-lophotrochozoan, with a secondary modification in the lophophorates. At present, such evolutionary deductions remain speculative, however, due to the lack of a reliable phylogeny for internal lophotrochozoan relationships and the unresolved question concerning the last common lophotrochozoan and bilaterian ancestor.

## Authors' contributions

JM performed research and drafted the manuscript. TW acquired the study material and helped with immunostaining and confocal microscopy. BL contributed to data interpretation. AW designed the study, supervised research, contributed to data interpretation, and improved the manuscript draft. All authors contributed to and approved the final version of the manuscript.
